# Highly Reliable Multicomponent MEMS Sensor for Predictive Maintenance Management of Rolling Bearings

**DOI:** 10.3390/mi14020376

**Published:** 2023-02-02

**Authors:** Elia Landi, Andrea Prato, Ada Fort, Marco Mugnaini, Valerio Vignoli, Alessio Facello, Fabrizio Mazzoleni, Michele Murgia, Alessandro Schiavi

**Affiliations:** 1 Department of Information Engineering and Mathematical Sciences, University of Siena, 53100 Siena, Italy; 2 Division of Applied Metrology and Engineering INRiM, National Institute of Metrological Research, 10135 Turin, Italy; 3 Department of Mechanical and Aerospace Engineering, Politecnico di Torino, 10129 Turin, Italy

**Keywords:** multicomponent MEMS sensor, low-cost, traceability, broad-frequency band, IoT condition monitoring

## Abstract

In the field of vibration monitoring and control, the use of low-cost multicomponent MEMS-based accelerometer sensors is nowadays increasingly widespread. Such sensors allow implementing lightweight monitoring systems with low management costs, low power consumption and a small size. However, for the monitoring systems to provide trustworthy and meaningful data, the high accuracy and reliability of sensors are essential requirements. Consequently, a metrological approach to the calibration of multi-component accelerometer sensors, including appropriate uncertainty evaluations, are necessary to guarantee traceability and reliability in the frequency domain of data provided, which nowadays is not fully available. In addition, recently developed metrological characterizations at the microscale level allow to provide detailed and accurate quantification of the enhanced technical performance and the responsiveness of these sensors. In this paper, a dynamic calibration procedure is applied to provide the sensitivity parameters of a low-cost, multicomponent MEMS sensor accelerometer prototype (MDUT), designed, developed and realized at the University of Siena, conceived for rolling bearings vibration monitoring in a broad frequency domain (from 10 Hz up to 25 kHz). The calibration and the metrological characterization of the MDUT are carried out by comparison to a reference standard transducer, at the Primary Vibration Laboratory of the National Institute of Metrological Research (INRiM).

## 1. Introduction

Nowadays, the management of monitoring and control for early fault diagnosis in equipment and industrial production processes is enormously enhanced, mainly thanks to the development of Machine Learning (ML) [1,2,3], Deep Learning (DL) [4,5] and Artificial Intelligence (AI) [6,7]. The possibility to manage huge data flows, in real time, from extensive sensors and multi-sensors networks [8], allows for monitoring the proper functioning of systems and sub-systems in detail [9,10], early identification of possible malfunctions [11], precisely localizing failures and planning proper decision making for targeted and timely intervention actions [12]. As highlighted in the cited review articles, high data quality is the premise and foundation of any functional and accurate monitoring process, in order to achieve the goal of the ‘Zero Defect Manufacturing paradigm’ [13].

The modernization and automation of the manufacturing industry involves the use of increasingly complex, interdependent (often interconnected), precise and fast mechanical equipment. The onset of a defect [14], if not properly identified [15], can cause cascading component failures [16], causing economic damage [17], financial losses [18] and industry downtime [19]. However, for the evolved ML and DL algorithms to provide reliable and trustworthy information on the monitored process, data provided by interconnected sensors must be truly accurate and traceable. Indeed, if data provided are incorrect or affected by uncontrolled bias or unknown errors, the learning system can store and return false or improper information. This, in turn, can generate a cascading effect of errors and therefore an inaccurate and unreliable diagnosis of the process to be monitored.

Metrological characterizations and suitable calibration procedures of sensors used in the monitoring networks are needed to guarantee the reliability of the measurements [20], providing high quality [21] and trustworthy information [22], ensuring a learning process based on reliable and accurate data [23]; moreover, in the case of sensor substitution, the calibration of data would allow to maintain the metrological traceability from sensor-to-sensor, ensuring the continuity of data-flow trustworthiness. Nevertheless, at present, suitable metrological calibration methods (traceable to the SI, International Systems of Units, and supported by detailed uncertainty budgets), can be applied only to ‘measuring instruments’, but not to ‘MEMS-based sensors’, since standard procedures are unavailable, and thus proper sensitivity parameters cannot be accurately identified. This lack, which prevents a functional and safe development of the latest generation monitoring systems, can be filled by the recent progresses in metrological research applied to micro mechanical systems and components [24]. This issue has been properly addressed by the Consultative Committee for Acoustic, Ultrasound and Vibration (CCAUV) of the Bureau International des Poids et Measures (BIPM), since «the deployment of such systems need to be underpinned by new metrology to support reliable and safe operation» of these technologies [25].

In this work, a tailored calibration procedure is developed and applied to provide traceability in the frequency domain (from 10 Hz up to 25 kHz) to a robust, lightweight, low-cost and low-power consuming multicomponent MEMS accelerometer sensor (MDUT), conceived for vibration monitoring of rolling-bearings. The MDUT is built by orthogonally embedding three separated uniaxial analogue sensing elements in a resin cube. The sensing elements are commercially available, low-noise, high frequency, uniaxial MEMS accelerometers, provided by Analog Devices Inc. (ADXL1005Z) [26]. The sensing elements independently detect the vibrations along three orthogonal axes, without cross-talk effects and transverse overlap. Although the manufacturer provided a calibration statement for individual ADXL1005Z MEMS sensor accelerometers, no information about traceability and related uncertainties is made available. In the following, the calibration of the whole assembled MDUT (realized by embedment of three sensing elements) is performed, in order to identify the main sensitivities and the transverse sensitivities along the three axes and the related uncertainties. Once the MDUT was opportunely calibrated, according to metrological standard procedures, the information provided of the dynamic phenomena under investigation is expected to be reliable and accurate enough to be managed, processed and returned by DL and AI systems, with the due trustworthiness.

## 2. Vibration-Based Rolling Bearings Fault Diagnosis: A Brief Survey

Since 1934, when the classic book by J. P. Den Hartog “*Mechanical Vibrations*” was first published [27], a very huge scientific and technical literature has amassed about rolling bearings fault diagnosis and functional monitoring in mechanical industry and manufacturing (namely, about 60,000 papers, according to the public Google Scholar database). In particular, during the last decades up to present day, thanks to the development of computational capabilities (e.g., ML, DL, AI and neural networks) and to the miniaturization of sensors and measuring systems (e.g., MEMS and NEMS), the management of early fault diagnosis, functioning monitoring, anomaly detection, as well as overall quality control procedures, has been greatly enhanced, with perspectives of further improvements in the near future.

In very general terms, applied procedures and methods for monitoring and early fault diagnosis of rolling bearings and related industrial processes are mainly supported by time–frequency–amplitude analysis of occurring vibrations [28,29,30,31], occasionally integrated with information on temperature fluctuations [32,33], noise levels [34,35] and other degradative harsh working environmental conditions affecting the functionality [36,37,38,39].

**Figure 1:**
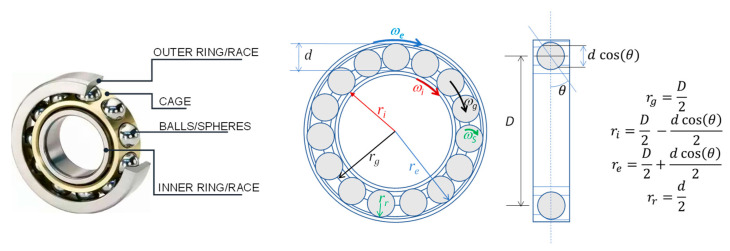
The basic elements of a rolling bearing and the related geometric parameters.

Rolling bearings are composed of four basic elements: the outer ring/race, the inner ring/race, a cage/train (to reduce friction and wear) and balls (or cylindrical rollers, spherical rollers, tapered rollers and needle rollers). Rolling-element bearings can range from ten millimeters to a few meters in diameter, with a load-carrying capacity from a few tens of grams to many thousands of tons. In Figure 1 a schematic picture of a typical rolling bearing and related elements and geometry are shown.

**Figure 2:**
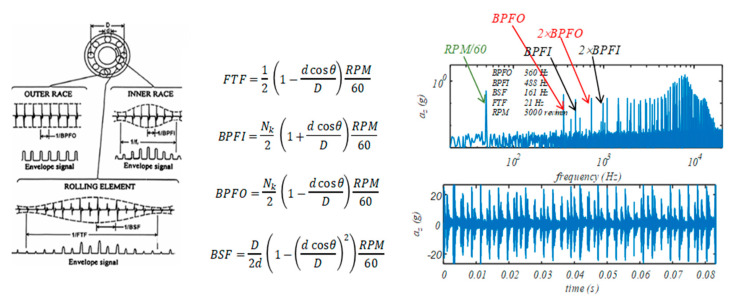
Example of characteristic rolling bearings frequency, and a typical time–frequency analysis. Left figure from [40].

Once the geometrical parameters and shape of the rolling bearing elements, the number of rotating balls/rollers (Nk) and the applied rotation velocity (RPM) are known, it is possible to univocally define the characteristic frequencies of the cage rotation (FTF), the ball passing at a given point of the inner race/ring (BPFI), the ball passing at a given point of the outer race/ring (BPFO) and the ball/roller spin (BSF), on the basis of simple formulae. Obviously, these formulae hold if no slips occur between the bearing elements. If a fault occurs, it can be related to the presence of a defect in one of the elements forming the rolling bearing, i.e., the inner ring/race, the outer ring/race or one ball. The rotation of the elements causes periodic impacts of the defect with the other elements, which in turn generate transient vibrations of the bearing components. Therefore, the vibrational signals can be seen as signatures of the different types of faults as it is a train of vibration transients with fundamental frequencies and spectral characteristics related to the location of the fault. In Figure 2, on the left, typical fault signatures are shown and correlated to the characteristic rotation frequencies of the elements of a rolling bearing (reported and defined in the figure). In the same figure, on the right, a vibration signal generated by a double fault (inner race and outer race) is plotted both in frequency (upper plot) and in the time domain (lower plot).

As shown in Figure 2, the characteristic rotation frequency values can be considered as a well-defined signature of the faulty element and can be clearly identified and monitored; any variation occurring in returned spectra, in terms of onset of harmonics, amplitude, width of peaks, clipping, as well as many other time–frequency–amplitude descriptors, are indications of incoming failures. In particular, frequency domain analysis of the signal or of the preprocessed signal (typically envelope extraction) involves extraction of features that can be found in a particular frequency band, allowing to identify localized or distributed bearing defects by means of spectrum analysis. Time domain analysis allows detecting defects and assessing their related magnitude by means of several statistical indicators, such as the peaks, peak-to-peak, energy content (Root Mean Square—RMS), the crest factor (CF), kurtosis (KU), skewness or the energy index (EI). In addition, combined time–frequency domain analysis allows capturing the progressive changes in spectrum components of the vibration signals in both frequency and time [40,41,42,43]. More recently, these classical methods have been replaced or reinforced by AI or ML techniques, used to identify defects and cope with noise or reduced measurement system bandwidth.

**Figure 3:**
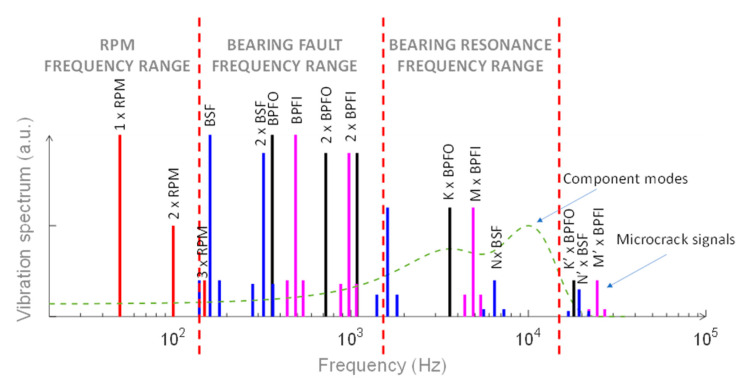
Spectral components of vibration signals due to a fault occurring in rolling bearings, with related frequency regions.

The earliest detectable indications of rolling bearings malfunctioning appear at very high frequencies, also beyond 20 kHz. At this early stage of deterioration, microscopic cracks appear, creating elastic waves with very low energy content. Once the deterioration progresses, the level of high frequency “noise” increases and the intensity of the impact forces excitation of the natural frequencies of the various bearing components; typically, depending on the rolling bearings dimensions and mounting, the natural frequencies range between 2 kHz and 6 kHz. When degradation increases, some sidebands above and below natural frequency resonances appear. After these early signals, the process of deterioration of the rolling bearings moves towards an irreversible damage and total failure. During this process, the clearances within the bearing increase substantially, and as a result, the overall vibration velocity amplitudes noticeably increase, the discrete bearing defect frequencies disappear and random vibrations appear in the form of a background noise. At this stage, total catastrophic failure of the machine can occur at any time. In Figure 3, an explicative summary of a typical spectrum of incoming failure in rolling bearings is shown.

In depth and accurate amplitude–time–frequency analysis of the rolling bearings signatures allows to properly identify the process of deterioration of the elements and the location and the typology of defects. Nevertheless, the accuracy of the analysis strongly depends on the quality of occurring vibrations measured quantities. The accuracy and the reliability of the sensors thus plays a crucial role in order to provide trustworthy information to be processed by the ML algorithms, as well as other computational processes.

## 3. Materials and Method

### 3.1. The MDUT and Embedded Sensing Elements

**Figure 4:**
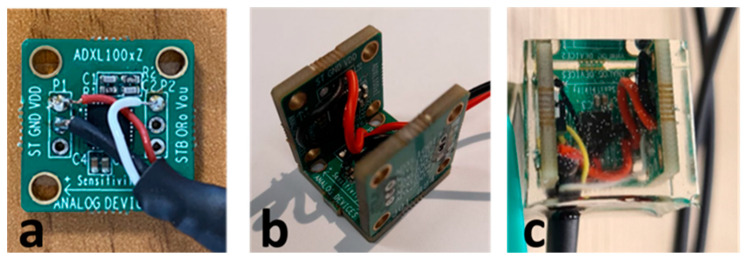
The MDUT elements: (**a**) single sensing element board, (**b**) multicomponent assembly, (**c**) epoxy encapsulation.

The MDUT is a multicomponent MEMS accelerometer sensor, realized at the University of Siena. Three individual sensing elements are orthogonally embedded in an epoxy resin cube. The MDUT is designed to be not very pervasive in terms of mass and size (it is a 30 g cube with sides of 2.5 cm). The sensing elements embedded in the MDUT are low-noise, high frequency uniaxial MEMS accelerometers, provided by Analog Device Inc. (Analog Devices, Norwood, London, England, MA, ADXL 1005Z), as shown in Figure 4.

The sensing element (ADXL 1005Z), according to the manufacturer’s information, has a frequency response from 0 Hz (DC) to 23 kHz (3dB frequency), with a resonant frequency at 42 kHz. The linearity is within ±0.1% of the full-scale range, with a cross axis sensitivity of ±1% along the perpendicular axes. The low-noise density is 30 µg/√Hz, it has a low-power consumption (current adsorption is 1.0 mA with 5 V power supply) and the single-supply operation is from 3.3 V to 5.25 V. The three sensing elements are installed on three specific demo boards, mounted by orienting the sensing axes orthogonally. The boards were initially fixed together with cyanoacrylate glue then they were coated with commercial epoxy resin.

### 3.2. Calibration Procedures

**Figure 5:**
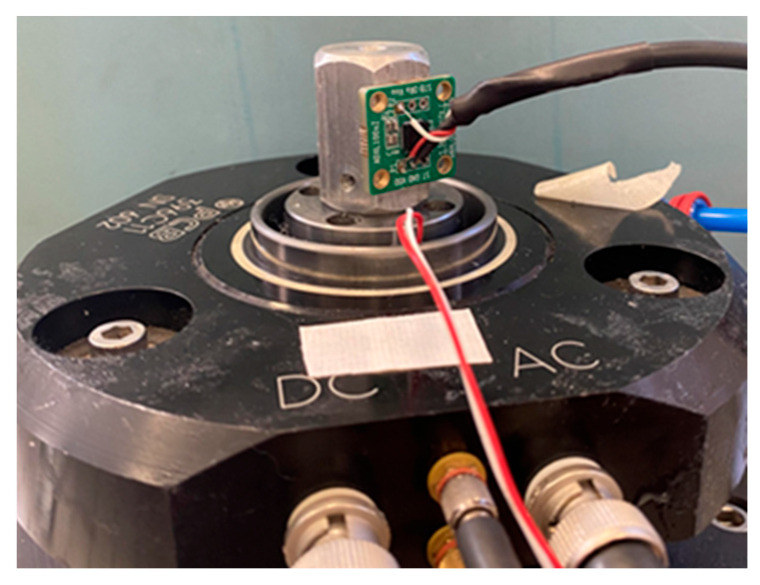
The individual sensing element ADXL 1005Z on the vertical instrumented vibration table.

As a first step, the sensing element ADXL 1005Z was individually calibrated to evaluate the intrinsic responsiveness as a function of deterministic mechanical vibrations. Calibration was carried out by gluing (Loctite^®^) the sensing element on a hexagonal prism mass and the assembly was screwed on a vertical instrumented vibrating table (PCB Piezotronics, Depew, NY, USA), as shown in Figure 5.

The instrumented vibration table was equipped with a single axis reference transducer (PCB Piezotronics, Depew, the United States, NY model 080A199/482A23), calibrated according to ISO 16063-11:1999 [44] against INRiM primary standard and integrated within the stroke of the shaker, allowing to measure the reference acceleration along the vertical axis, aref (parallel to g). The calibration of the individual sensing element was carried out according to ISO 16063-21:2003 [45]. The reference acceleration was acquired by an acquisition board NI 4431 (National Instruments, Austin, TX, USA) with a sampling rate of 50 kHz, fed to the PC and processed through LabVIEW^®^ 22 software to provide the RMS reference value in ms^−2^. Actually, the calibration of the individual sensing element, by using the set-up shown in Figure 5, was affected by some systematic errors induced by the vibrational modes of the hexagonal prism mass, and by the small (but not negligible) damping of the glue. The errors depend on both frequency and amplitude and cannot be experimentally evaluated with this calibration setup; thus, it needs to be considered in the evaluation of the overall uncertainty [46,47,48].

**Figure 6:**
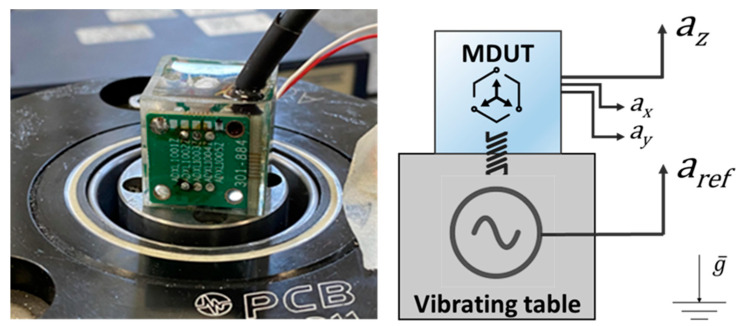
The vertical axial calibration of the MDUT embedded in the resin case: the MDUT on the instrumented vibrating table and a schematic picture of the calibration assembling.

Once the sensitivity of the individual sensing element was known, the calibration of the MDUT, embedded in resin, was carried out. Calibration was performed by screwing the MDUT directly onto the vibrating instrumented table, as shown in Figure 6. The aim of the investigation was to characterize the possible effects on the sensing elements response due to the embedding resin and to quantify the occurring transverse accelerations in the horizontal direction.

### 3.3. Calibration Results

**Figure 7:**
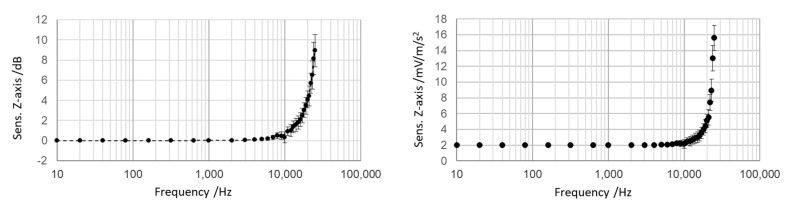
Sensitivity expressed in mV/ms^−2^ and in dB of the individual sensing element.

Calibration was carried out from 10 Hz up to 25 kHz and the sensitivity values were determined with different values of power supply, namely 3.5 V, 4 V, 4.5 V and 5 V. Since the standard deviation of calibration at different values of power supply was negligible (<1%), the overall average values were considered representative of the actual sensitivity of the individual sensing element under investigation. In the graphs in Figure 7, the sensitivity of the individual sensing element ADXL 1005Z with associated uncertainties is shown. Data expressed in dB are referred to as the sensitivity at 10 Hz, which is 2.004 mV/(ms^−2^).

Sensitivity values were determined with the calibration set-up shown in Figure 5. By applying this procedure, it could be seen that the 10% accuracy bandwidth is 12 kHz. The effect of the intrinsic resonance (at 47 kHz, according to the manufacturer’s information) amplifies the sensitivity of 3 dB to 18 kHz (slightly lower than the typical value provided by the manufacturer’s specifications) and 6 dB to 21.5 kHz. 

**Figure 8:**
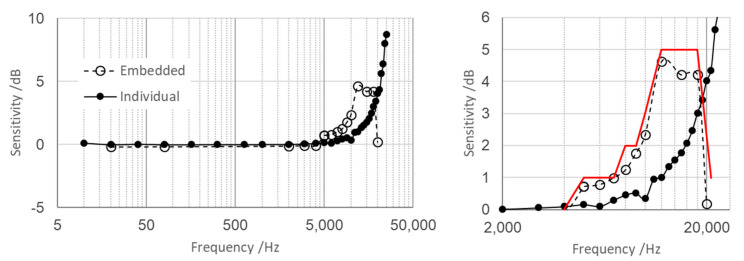
Full scale range (**left** plot) and high-frequency range detail with related calibration uncertainties (**right** plots) of the comparison between the sensitivity of the individual sensing element and the sensitivity of the embedded sensor along the vertical axis.

The sensitivity of the vertical single axis of the MDUT was then determined by screwing the resin case onto the instrumented vibrating table. Calibration was performed according to the set-up shown in Figure 6, from 10 Hz up to 25 kHz, and the sensitivity values were compared with the sensitivity of the individual sensing element not embedded in resin. Figure 8 shows a comparison of the calibration experimental results in the whole tested frequency range in the left-hand plot, and in detail between 2 kHz and 25 kHz in the right-hand plot.

As observed, the effects due to the resin of the embedding case affect the amplitude of the frequency response of the sensing element with respect to the individual calibrated sensor. In particular, the embedding case acts as a “damper” (or mechanical low-pass filter) by smoothing the intrinsic resonance peak. The amplification due to the intrinsic resonance of the embedded sensing element was limited to 2 dB up to 10 kHz, and increased to 5 dB at 20 kHz. Beyond 21.5 kHz, the external vibrations were fully dampened. Once the amplification was quantified, a proper correction factor can be applied, e.g., between 5 kHz and 20 kHz, allowing to provide accurate measurements of the vibration amplitude in the typical frequency range of bearing resonance frequencies, with the related onset of sidebands near the resonance and the onset of random vibration noise, as shown in Figure 3.

**Figure 9:**
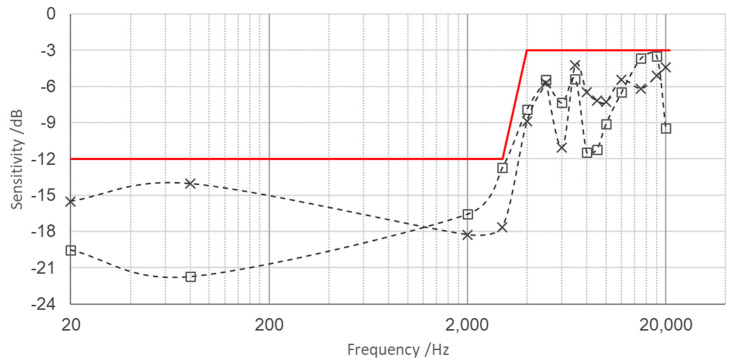
Full scale range of the relative amplitude of transverse sensitivity (grey dotted lines, square X axis, cross Y axis) on the horizontal plane with respect to the sensitivity measured along the vertical axis, which is the direction of reference acceleration (parallel to *g*).

The following graph in Figure 9 shows the measured relative transverse sensitivities on the horizontal plane (along the x and y directions) by the sensing elements embedded perpendicularly with respect to the vertical direction (z) of the applied dynamic acceleration. The transverse sensitivities can be considered negligible up to 14 kHz, where the amplitudes values are in general below −6 dB and around −3 dB up to 25 kHz.

### 3.4. Metrological Characterization

**Figure 10:**
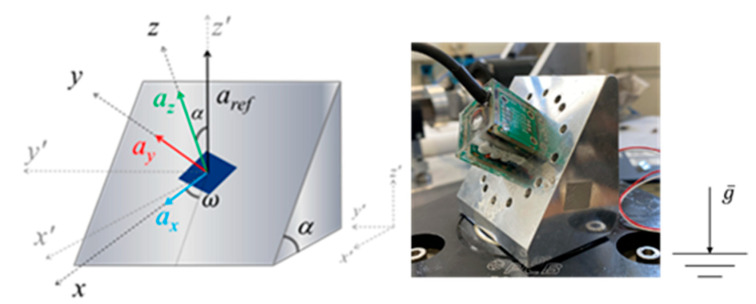
The multicomponent calibration system: inclined plane allows to generate a projection of the vertical reference acceleration along three axes simultaneously.

The main sensitivities along the three axes were also evaluated in order to define the actual responsiveness of the MDUT in dynamic conditions. Once the sensitivity of the vertical axis of the individual sensing elements embedded in the resin was known, the MDUT was mounted on an inclined plane screwed on to the vibrating table. This experimental set-up allowed to generate a projection of the vertical reference acceleration, *a_ref_*, along the three axes simultaneously. In this application, a specific system suitable for the simultaneous amplitude calibration of multicomponent MEMS sensors was used. The calibration method and the experimental set-up, shown in Figure 10, have been described in detail in [49,50].

By means of simple geometrical laws, it is possible to define the expected acceleration along the three main axes as follows:

(1) $$\left\{\begin{array}{c} a_{x}=\left|a_{ref}\sin\left(\alpha \right)\cos\left(\omega \right)\right|\\ a_{y}=\left|a_{ref}\sin\left(\alpha \right)\sin\left(\omega \right)\right|\\ a_{z}=\left|a_{ref}\cos\left(\alpha \right)\right| \end{array}\right.,$$

where α is the tilt angle (*α* = 55° ± 0.1°) and ω is the rotation angle (*ω* = 45° ± 0.1°).

A nearly constant peak amplitude of 1 m/s^2^ was applied between 100 Hz and 1 kHz and the corresponding experimental sensitivities were determined according to the inclination and rotation angles. Any difference observed between the experimental values of acceleration along the three axes and the expected values of acceleration, from Equation (1), were dependent on both the possible differences in individual sensitivities of the embedded sensors and on possible misalignment due to the gluing and embedding of the sensing elements in the resin cube. Once these differences were known it was possible to definitively adjust the sensitivities of the MDUT. 

**Figure 11:**
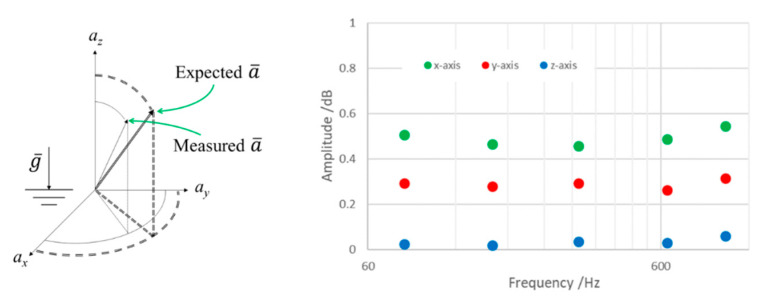
Quantification of differences between the measured acceleration and expected acceleration.

As shown in Figure 11, by measuring the occurring accelerations along the three axes of the MDUT on the inclined plane during calibration, and by calculating the difference between the expected acceleration (from Equation (1)) and the measured ones, the possible misalignment among the three sensitivity axes, as well as the possible differences in the proper sensitivities of the sensing elements orthogonally glued and embedded in the resin cube, were quantified. These differences can be considered as systematic effects to be adjusted.

As is possible to notice, very small differences were observed between measured and expected values of MDUT sensitivities. This means that systematic effects due to possible misalignments among sensing elements and differences among the proper sensitivities were negligible, at less than 1 dB. The complete description of the method to be applied for calculation can be found in [51]; in this application the misalignment was assessed in dynamic conditions in the gravity field instead of in static conditions.

### 3.5. The Role of the Embedding Resin Case: Actualization of a “Mechanical Filter”

**Figure 12:**
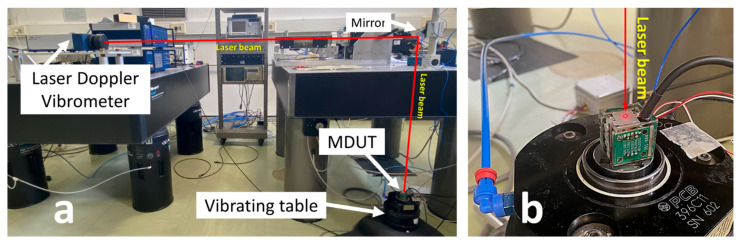
Characterization of the mechanical behavior of the embedding resin case during MDUT calibration in dynamic conditions: (**a**) the whole measuring system and (**b**) the MDUT on the vibrating table hit by the single-point Laser-Doppler vibrometer beam on the upper surface.

The frequency response of the embedded sensing element was “dampened” by the mechanical dissipative behavior of the epoxy resin, as shown in Figure 8. As a matter of fact, the cube (in which sensing elements are encapsulated) acted as a mechanical filter [52,53,54] due to the intrinsic elastic and dissipative properties of the constitutive polymeric material. In order to quantify the mechanical behavior of the embedding resin case, once subjected to vertical acceleration, the actual vibrations occurring on the MDUT during calibration were monitored by single-point Laser-Doppler velocimetry (Laser head Polytec GmbH, Waldbronn, Germany, model OFV 505, vibrometer controller Polytec GmbH, Waldbronn, DE, model OFV 5000), acquired by an acquisition board NI 4431 (sampling rate of 50 kHz) integrated in a PC and processed through LabVIEW^®^ software. The laser beam hits the upper surface of the MDUT, returning the frequency response of the resin case, as shown in Figure 12.

**Figure 13:**
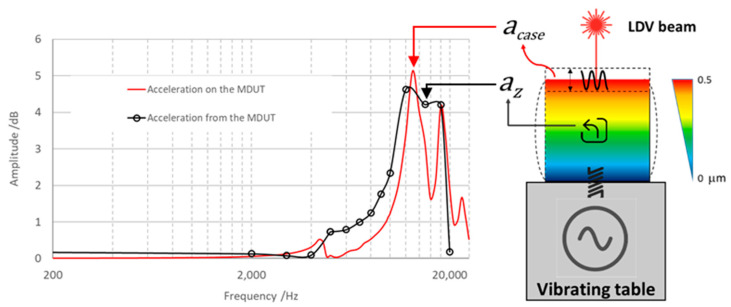
Comparison between the acceleration amplitude, *a_case_*, measured on the upper surface of MDUT by the Laser-Doppler Vibrometer (red curve), the acceleration amplitude, *a_z_*, measured from the signal output of the sensing element embedded in the resin case (black curve) and a schematic picture of the mechanical behavior and deformations occurring during calibration.

The frequency response, measured on the upper surface of the MDUT, allows to evaluate the mechanical behavior of the epoxy resin case during dynamic vertical excitation. In the graph in Figure 13, the comparison between the relative amplitude of acceleration measured by the sensing elements within the resin case, *a_z_*, and the corresponding amplitude of acceleration measured on the resin case, *a_case_*, along the vertical axis with respect to the reference acceleration amplitude, *a_ref_*, are represented.

As it is possible to notice, the acceleration amplitude measured by the sensing element embedded in the resin case was modelled according to the resonant modes of the resin cube. This mechanical effect has two very useful practical advantages which can be favorably exploited in the early fault diagnosis monitoring process: the deep attenuation of the sensor resonance and the mechanically controlled amplification of the signal in a well-defined frequency range. Indeed, since the amplification due to the intrinsic resonance frequency of the free sensing element rapidly increases, according to the slope of sensitivity shown in Figure 7, serious waveshape distortions could occur in the frequency range starting from 0.1 times the resonance frequency (namely from 4.7 kHz), and more relevant measurement uncertainties are expected beyond 12 kHz, where the accuracy of the bandwidth exceeds 10%. Once the amplitude of the intrinsic resonance of the sensing element is dampened, the frequency range exploitable for measurements can be extended up to 25 kHz or beyond, depending on the measurement capabilities. Moreover, since the mechanical amplification occurs in a well-defined frequency range, with a known quality factor and peak amplitude, it is possible to exploit this effect to identify the onset of microscopic cracks beyond 20 kHz and up to 25 kHz early, by considering the amplification as kind of a “magnifying glass” pointed at a certain frequency range. In consequence, it is possible to opportunely design the embedding case with different resins in order to build up tunable mechanical filters and amplifiers, depending on the frequency range to be monitored. 

**Figure 14:**
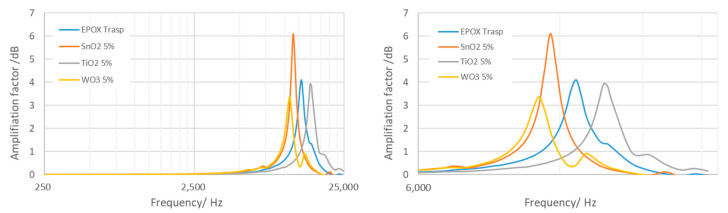
Mechanical behavior of four resins subjected to dynamic excitation. The resonant frequency peaks are clearly identified, as well as the quality factor and amplification factor of each sample.

In the following, a comparison between the mechanical behavior of four different embedding resins (including only epoxy) is carried out by using the method and the procedure described above. In this analysis, only the embedding resin material is investigated in order to identify the performance of different types of materials. To this aim, four cubes were characterized, the first of which was bare epoxy resin, whereas the other three were obtained by reinforcing the resins with nanoparticles of SnO_2_ (5% *v*/*v*), TiO_2_ (5% *v*/*v*) and WO_3_ (5% *v*/*v*). The comparison is shown in the graph in Figure 14.

The experimental characteristics of the embedding resins resonant modes are summarized in Table 1. These parameters allow to define the properties of the cases acting as mechanical filters and amplifiers.

## 4. Reinforced Resins: MDUTs Realization and Test through Case Studies

From the experimental characterization of the different reinforced resins, it is possible to notice that the epoxy filled with 5% (*v*/*v*) TiO_2_ is the one with the highest resonant frequency. A second embedded MDUT was realized by exploiting this reinforced resin.

The realization follows the same procedure as the one used for the MDUT in bare epoxy, adopting three ADXL1005 MEMS accelerometers in a triaxial configuration. 

Tests were performed exploiting the test bench described in [55], which adopted a modified loudspeaker as a vibration source. The actuator consists of a loudspeaker magnetic motor and a moving assembly realized exploiting the speaker voice coil. The MDUT and a reference accelerometer were mounted on an aluminum disc firmly fixed at the edge of the voice coil. 

The responses of the two MDUTs were compared with the one of a triaxial piezoelectric reference accelerometer (B&K, Nærum, Denmark, model 4426) characterized by 10% accuracy bandwidths of 9 kHz, 8 kHz and 16 kHz along the x, y and z axes, respectively. The z-axis was mounted in the direction of the aluminum plate displacement. All the accelerometers were tested by screwing them on to the aluminum plate with an M3 thread.

For all the accelerometers, the signals of the three axes were acquired with an acquisition board (National Instruments, Austin, TX, USA, model DAQ PCI 6259 1.2 MS/s, 16 channels, 16 bits) with a sampling frequency of 160 kHz.

To test the DUTs in realistic conditions, excitation signals emulating the bearing fault vibration signatures were chosen as excitation signals with a harmonic content up to 50 kHz; the bandwidth of a power amplifier equal to 100 kHz. In this context, the loudspeaker acts as a mechanical filter for the higher frequencies. Tests performed with the reference accelerometer proved that the harmonic content of the acceleration generated by exciting the loudspeaker with the emulated signals was extended up to 20 kHz. The reported results are the responses of the two MDUTs and the piezo reference accelerometer along the excitation axis (z axis).

The devices were tested individually since the emulated signals were stationary and the payload given by the two different devices was comparable in terms of added mass.

In particular, vibration signatures of different types of single faults, i.e., outer race, inner race and ball faults, were emulated exploiting the technique presented in [56], based on the following model for the vibration signature, *s(t)*:

(2) $$s\left(t\right)=\left(1+K_{F}\sin\left(\frac{2\pi f_{F}}{60}\right)\right)\overset{}{\underset{i}{\sum }}[\frac{A_{C1}}{2}\left(\;1-{\left(-1\right)}^{i}\right)+\frac{A_{C2}}{2}\left(\;1-{\left(-1\right)}^{i+1}\right)e^{-\frac{t-\frac{i}{F_{C}}}{\tau }}\sin\left(2\pi f_{res}\left(t-\frac{i}{F_{C}}\right)\right)\;u\left(t-\frac{i}{F_{C}}\right)\;+A_{RPM}\sin\left(\frac{2\pi RPM}{60}\;t+\phi \right)$$

where the first term of the sum is the acceleration signal due to the presence of the fault, given by a train of transient vibration of the bearing components started by each defect impact, and *u(t)* is the step function with *u(t)* = 1 if *t ≥* 0, *u(t) =* 0 if *t* < 0. *F_C_* = *BPFO* in the case of an outer race fault, *F_C_ = BPFI* in the case of an inner race fault and *F_C_ = BSF* in the case of a ball fault. *A_C_*_1_ *= A_C_*_2_ *= A*_0_ and *K_F_ =* 0 in the case of an outer race fault; *A_C_*_1_ *= A_C_*_2_ *= A*_0_, *K_F_ ≠* 0 and *f_F_ = RPM* in the case of an inner race fault; and *A_C_*_1_ > *A_C_*_2_*, K_F_ ≠* 0 and *f_F_ = FTF* in the case of a ball fault. *f_res_* is the resonance frequency of the bearing components, τ is the time constant characterizing the component transient vibration and *A_RPM_* is the amplitude of the component related to the shaft rotation. The leftmost term in Equation (2) represents the vibration of the accelerometer due to the vibration of the rotating part of the engine and can be seen as a low frequency noise. 

Obviously, this mathematical model of bearing failures holds if no slipping occurs in the bearing components. In real applications, this is true when the failures are at early stages and the mechanical load on the bearing itself is not critical. When the defect increases in severity, slipping starts to occur, especially in the case of ball failures. Moreover, in real life, the accelerometers can be mounted far from the bearings or shafts; in this case, the vibration components, which are present near the bearing structure, can be attenuated by the path between the vibration source and the sensor itself and disturbed by other vibration signatures generated by other mechanical sources.

**Figure 15:**
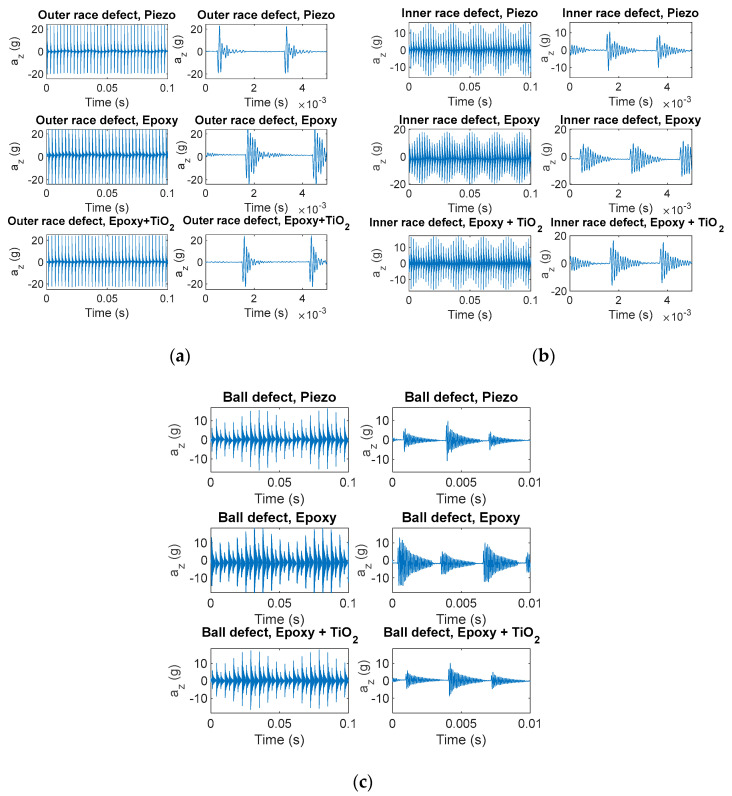
Measured fault signature vibration signals in the time domain. Sampling frequency = 160 kHz. (**a**) Outer race fault; (**b**) inner race fault; and (**c**) ball fault. Upper plots—signals measured by the reference piezoelectric accelerometer; middle plots—signals measured by the proposed MDUT encapsulated in epoxy resin; and lower plots—signals measured by the proposed MDUT encapsulated in epoxy resin filled with TiO_2_.

**Figure 16:**
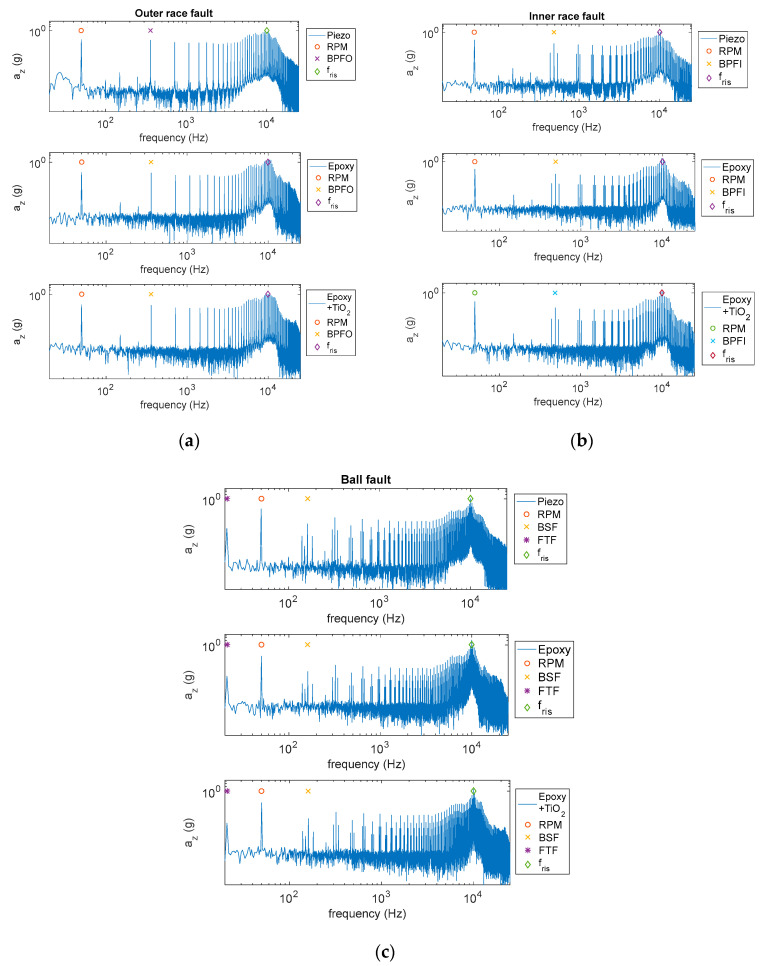
Measured fault signature vibration signals spectra. Sampling frequency = 160 kHz. (**a**) Outer race fault; (**b**) inner race fault; and (**c**) ball fault. Upper plots—signals measured by the reference piezoelectric accelerometer; middle plots—signals measured by the proposed MDUT encapsulated in epoxy resin; and lower plots—signals measured by the proposed MDUT encapsulated in epoxy resin reinforced with TiO_2_.

Many different conditions were considered, accounting for different roller bearing types, machine RPMs and component resonant frequencies, *f_res_*, and in all cases the performance of the MDUTs proved to be satisfactory in accordance with what was expected from the calibration study. Resonance frequencies up to 10 kHz were considered. The following figures show some examples of the results obtained considering a roller bearing (22209E1K bearing), an RPM = 3000 rev/min and resonant frequency of the bearing components, *f_res_* = 10 kHz, by emulating an outer race fault characterized by BPFO = 361 Hz, an inner race fault with BPFI = 488 Hz and finally a ball fault with BSF = 161 Hz and FTF = 21 Hz. In detail, Figure 15 presents three different cases of fault signature vibration signals in the time domain, whereas Figure 16 shows the same signals in the frequency domain. As it can be seen, both the presented MDUTs allow for an accurate detection of faults in both the time and frequency domains, even considering the high frequency band of the component resonant (modal) vibrations emulated in this test. As expected, reinforcing the epoxy resin with TiO_2_ provided a larger bandwidth and a higher fidelity.

## 5. Conclusions

In this paper, we present a low-cost, multicomponent MEMS sensor accelerometer prototype (MDUT), designed, developed and realized at the University of Siena, to be applied for rolling bearings vibration monitoring in a broad frequency domain. The technical performance of the MDUT is evaluated from calibrations and suitable metrological characterizations at the Primary Vibration Laboratory of the National Institute of Metrological Research (INRiM). In particular, both the sensitivity of the individual sensing element and the sensitivity of the MDUT embedded in resin are determined, according to ISO 16063-21:2003. Calibration of MDUT is carried out from 10 Hz up to 25 kHz, and the main sensitivities along the three axes are evaluated in order to define the actual responsiveness of the MDUT in dynamic conditions; this approach allows to identify and quantify possible misalignments among the axes and to quantify the occurring transverse accelerations and cross-sensitivities. Moreover, the mechanical behavior of the embedding resin case, once subjected to vertical acceleration, is determined in order to evaluate the actual vibrations occurring on the MDUT during operative conditions. Since it is observed that the resin cube acts as a “mechanical filter”, four different kinds of embedding resins; namely, epoxy resin alone and epoxy resins filled with nanoparticles of SnO_2_ (5% *v*/*v*), TiO_2_ (5% *v*/*v*) and WO_3_ (5% *v*/*v*), are investigated and their mechanical behaviors compared. The epoxy filled with 5% (*v*/*v*) TiO_2_ is the one with the highest resonant frequency; therefore, a MDUT embedded with this reinforced resin is applied for case studies to test the DUTs in realistic operative conditions. Tests were undertaken exploiting a previously realized test bench which allows to emulate different vibration signatures of bearing failures considering the different bearing characteristics. As expected, the device realized with reinforced epoxy performs better than the one realized with epoxy alone in terms of fidelity of the reproduction and bandwidth.

## Figures and Tables

**Table 1:** Characteristics of the mechanical filters.

Resin Case	Resonant Frequency /kHz	Quality Factor /-	Relative Amplification Factor /dB
Epoxy only resin	13.1	3.0	4
Epoxy with 5% SnO_2_	11.5	13.1	6
Epoxy with 5% TiO_2_	15.2	2.6	4
Epoxy with 5% WO_3_	10.8	2.4	3.5

## Data Availability

Data sharing not applicable. No new data were created or analyzed in this study. Data sharing is not applicable to this article.
